# Snake Bites—Treated by Brandy

**Published:** 1855-07

**Authors:** J. R. Thomas

**Affiliations:** Monticello, Ind.


ARTICLE IV.—*Snake Bites—Treated by Brandy.* By J. R. Thomas, of Monticello, Ind., April, 1855.

You will please pardon me for troubling you with a few remarks on the subject of snake bites. I have been practising in N. W. Indiana for some twelve years, and in this time have been called to visit many patients suffering from the bites of numerous kinds of poisonous serpents. My practice has been to cup the wound freely, if practicable; if not, use suction with the mouth. While operating in this way, I administer brandy, or proof spirits of any kind, in large and frequent doses, and recommend my patients to become intoxicated, if possible; and furthermore, I recommend the use of some mild cathartics. Never have I been compelled to visit my patients more than twice, and often only once.

In August, 1852, I was sent for in great haste to visit Mr. J. H. S., (Age 38 years, who stands six feet six in his stockings; who, by the way, was very fond of brandy,) who had just been bit on the inside of his left heel by a large rattle snake,—both fangs had been well inserted in the muscles. I treated him as above in 36 hours—wounds sound and well. I gave him in the short time alluded to, 1 quart brandy and 1½ gallons whiskey—all without any intoxication. He wanted more, and I refused to supply his wants.

The next day Mr. H., his next neighbor, was passing along by Mr. J. H. S., and saw him with his pants rolled to his knees and barefooted, wading round in some weeds and grass feeling with his feet. Mr. H. asked him if he had lost anything. Answer —no sir. What are you doing there then? I am hunting a snake. There ain't any liquor only what Doc. Thomas has, and he wont let me have any unless I am snake bit, so I am hunting one.

---

Remarks by Ed.—We publish the above not for any intrinsic value which it possesses, but because it affords an interesting example of the *strength* of a man's appetite for *strong drink* on the one hand, and a good specimen of the imperfect record of facts on the other. That any human being should have his nervous system so habituated to the stimulus of Alcohol, that he can take *one quart* of brandy and *one* and *a half gallons* of whiskey in the space of 36 hours, without intoxication, is only equalled by the fact that his appetite for the liquor should be so strong as to cause him to deliberately seek a rattle-snake bite for the sake of more liquor.

But the facts connected with Dr. Thomas's experience in the treatment in snake bites are so imperfectly stated as to destroy entirely their practical value. He reports uniform success with the internal administration of Alcoholic drinks; but he omits altogether to state, at what stage in the symptoms of poisoning he commences their use, or even whether any general symptoms of actual poisoning had occurred at all. To judge correctly of the efficacy of any particular mode of treatment, it is necessary to know the symptoms at the time the medicine is given; simply because it is well known that many such snake bites occur which result in no serious poisoning, when no remedies whatever are used. This fact alone renders the foregoing communication, desitute as it is of all details, comparatively worthless. We receive many such, and our present comments are not so much aimed at this particular paper, as at the frequent practice of *reporting* observations in general terms. The day has gone by for these loose generalizations,— Science requires both fullness and accuracy in all her observed facts.

